# Intervenciones de enfermería en la reversión del estoma intestinal: revisión integrativa*

**DOI:** 10.15649/cuidarte.2165

**Published:** 2022-08-27

**Authors:** Norma Yaneth Gómez Barriga, Mauricio Medina Garzón

**Affiliations:** 1 Enfermera, Magíster en Enfermería Universidad Nacional de Colombia Sede Bogotá, Facultad de Enfermería, Bogotá-Colombia. E-mail: nygomezb@unal.edu.co Universidad Nacional de Colombia Universidad Nacional de Colombia Facultad de Enfermería Bogotá Colombia nygomezb@unal.edu.co; 2 Profesor, Enfermero, Magíster en Enfermería, Universidad Nacional de Colombia Sede Bogotá-Facultad de Enfermería, Bogotá-Colombia. E-mail: mamedinaga@unal.edu.co Universidad Nacional de Colombia Universidad Nacional de Colombia Facultad de Enfermería Bogotá Colombia mamedinaga@unal.edu.co

**Keywords:** Cierre de estoma, Reversión de Estoma, Ileostomía, Colostomía, Enfermería, Stoma reversal, Stoma Closure, Ileostomy, Colostomy, Nursing, Fechamento do estoma, Reversão do estoma, Ileostomia, Colostomia, enfermagem

## Abstract

**Introducción:**

los estomas intestinales representan un impacto significativo en la calidad de vida de las personas; sin embargo, estos deben revertirse después de haberse restituido el tránsito intestinal o la resolución del proceso inflamatorio inicial. Por otro lado, la negación de la persona para su reversión puede deberse a la falta de información y orientación por parte de los profesionales de la salud. Por lo anterior es importante identificar las intervenciones de Enfermería en la atención de la persona con reversión del estoma intestinal.

**Materiales y métodos:**

se realizó una revisión integrativa de la literatura de alcance descriptivo en el período comprendido entre los años 2015 a 2020, a través de las bases de datos *Wos*, *Pubmed*, *Scopus*, *Scielo* y *Cochrane*. Se seleccionaron 36 artículos que cumplieron con los criterios de inclusión y exclusión con el respectivo análisis metodológico.

**Resultados:**

Se identificaron las siguientes intervenciones de Enfermería, para el preoperatorio: valoración preoperatoria, preparación intestinal y seguimiento a comorbilidades. El intraoperatorio: profilaxis, preparación de la piel, técnica quirúrgica y cierre de la pared abdominal. En el posoperatorio: cuidado de la herida quirúrgica, calidad de vida y educación.

**Discusión:**

es importante la reflexión sobre el tiempo de reversión, la técnica quirúrgica y la importancia de las intervenciones por Enfermería.

**Conclusión:**

Enfermería cumple un papel importante en la reversión del estoma, no solo por los cuidados físicos y la educación que se brinda, sino también en las intervenciones aplicables al contexto social y emocional que afectan el estilo de vida de la persona.

## Abstract (en)

**Introduction:**

Intestinal stomas have a significant impact on people’s quality of life. However, these should be reversed after gastrointestinal transit has been restored or the initial inflammatory response has gone. In addition, patients may refuse to reverse them due to a lack of information and guidance from healthcare professionals. Therefore, it is important to identify nursing interventions in caring for patients with stoma reversal.

**Materials and Methods:**

An integrative literature review was conducted in WOS, PubMed, Scopus, SciELO and Cochrane databases published between 2015 and 2020. 36 articles were selected based on inclusion and exclusion criteria with their methodological analysis.

**Results:**

The following nursing interventions were identified: preoperative assessment, bowel preparation and follow-up of comorbidities in the preoperative period; prophylaxis, skin preparation, surgical technique and abdominal wall closure in the intraoperative period; surgical wound care, quality of life and education in the postoperative period.

**Discussion:**

It is essential to reflect on reversal time, surgical technique and the importance of nursing interventions.

**Conclusions:**

Nursing plays an important role in stoma reversal not only because of physical care and education provided, but also the interventions applicable to social and emotional contexts affecting the patient’s lifestyle.

## Resumo

**Introdução:**

Os estomas intestinais representam um impacto significativo na qualidade de vida dos indivíduos; entretanto, eles devem ser revertidos após o trânsito intestinal ter sido restaurado ou o processo inflamatório inicial ter sido resolvido. Por outro lado, a recusa de uma pessoa em revertê-los pode ser devido à falta de informação e orientação dos profissionais de saúde. Portanto, é importante identificar intervenções da Enfermagem no cuidado da pessoa com reversão do estoma intestinal.

**Materiais e métodos:**

no período de 2015 a 2020 foi realizada uma revisão integrativa da literatura de escopo descritivo, através das bases de dados Wos, Pubmed, Scopus, Scielo e Cochrane. Foram selecionados 36 artigos que preenchiam os critérios de inclusão e exclusão com a respectiva análise metodológica.

**Resultados:**

Foram identificadas as seguintes intervenções de Enfermagem, para o período pré-operatório: avaliação pré-operatória, preparação intestinal e monitoramento de comorbidades. Intra-operatório: profilaxia, preparação da pele, técnica cirúrgica e fechamento da parede abdominal. Pós-operatório: tratamento de feridas cirúrgicas, qualidade de vida e educação.

**Discussão:**

é importante refletir sobre o tempo de reversão, a técnica cirúrgica e a importância das intervenções de Enfermagem.

**Conclusão:**

A Enfermagem desempenha um papel importante na reversão do estoma, não apenas nos cuidados físicos e na educação fornecida, mas também nas intervenções aplicáveis ao contexto social e emocional que afetam o estilo de vida da pessoa.

## Introducción

Los estomas intestinales son frecuentes en la práctica clínica y son comunes en personas con enfermedades de origen intestinal. Se estima que, por cada mil adultos, entre dos y cuatro individuos son portadores de una ostomía intestinal. El tipo de ostomía que más se realiza es la colostomía (55,1%), seguida de la ileostomía (35,2%) y el 65% son temporales que requieren reversión^1^.

El cierre o reversión consiste en extirpar el estoma existente, realizar una anastomosis y devolver el intestino a la cavidad abdominal^2^. Además, este procedimiento quirúrgico tiene una morbilidad entre el 10 y el 50% por los diversos riesgos preexistente en la persona y los factores de reversión a la intervención quirúrgica^3^; por otro lado, la incidencia anual de estomas intestinales aumenta de manera global debido al cribado y al tratamiento del cáncer colorrectal, así como a los avances técnicos y diagnósticos de enfermedades inflamatorias intestinales, diverticulitis y diferentes tipos de cáncer^4^.

Otra preocupación que surge son las consecuencias de la reversión del estoma que a menudo son subestimadas y generan un impacto en la persona. En el caso de las ileostomías, estas pueden presentar complicaciones como la obstrucción intestinal, infección de la herida quirúrgica, peritonitis, absceso intraabdominal, fugas anastomóticas, fístulas entero-cutáneas y sangrado en menor proporción que en las colostomías^5^. Por otro lado, la negación del paciente para su cierre ocasiona un retraso mayor a seis meses en la reversión del estoma, lo que aumenta las complicaciones y la duración de la estancia postoperatoria, así como la incrementación de los costos asociados a la atención y al cuidado^6^.

Las personas que se revierten se enfrentan a dos situaciones: a la reconstrucción del tránsito intestinal con sus potenciales complicaciones y al cierre de la pared abdominal que puede generar dificultades; sin embargo, este procedimiento depende de otros factores como la resolución del proceso inflamatorio inicial, la enfermedad de base y el estado general del paciente^7^. Asimismo, el tiempo de reversión más corto podría prevenir la atrofia del muñón rectal. Dentro de las principales causas del retraso se encuentran la quimioterapia adyuvante, la enfermedad de base y la fuga anastomótica. Del mismo modo, la edad del paciente y el rechazo son las principales razones para la no reversión^8^.

Es fundamental que el profesional de Enfermería reconozca su actuar en el cuidado de la reversión del estoma y el impacto que genera en la persona, así como es necesario aplicarlo de forma específica y personalizada en cada sujeto con la finalidad de atenuar el riesgo de complicaciones como de su incidencia y de su morbimortalidad^9^. Por lo anterior, es importante identificar las intervenciones de Enfermería en la atención de la persona con reversión del estoma intestinal mediante una revisión de la literatura.

## Materiales y Métodos

Se realizó una revisión integrativa de la literatura, de alcance descriptivo y retrospectivo, fundamentada específicamente en los parámetros establecidos por Whittemore y Knafl^10^, mediante el seguimiento de los lineamientos establecidos por PRISMA. Se consideraron cinco etapas: identificación del problema, búsqueda en la literatura de estudios primarios, evaluación de estudios, análisis de datos y presentación de resultados.

Etapa N.º 1: Identificación del problema

El problema abordado en la presente revisión se formuló a partir de la siguiente pregunta de investigación: ¿Cuáles son las intervenciones de Enfermería en la persona con reversión del estoma intestinal? Las condiciones que deben cumplir los estudios para participar en esta revisión fueron establecidas por medio de la identificación de la pregunta PICOT: **P:** Personas con ostomía intestinales (colostomía e ileostomía), **I:** Reversión del estoma intestinal, **C:** No aplica, **O:** Intervenciones de Enfermería, **T:** 2015-2020.

Etapa N.º 2: Búsqueda de literatura

Las estrategias de búsqueda de literatura estrictamente definidas son fundamentales para mejorar el rigor de cualquier tipo de revisión. Por lo tanto, para el desarrollo de esta etapa, se determinaron los siguientes parámetros: el tema, los descriptores de búsqueda y los criterios de inclusión y exclusión.

Dentro los criterios de inclusión se consideraron: los artículos publicados en el periodo comprendido entre los años 2015 a 2020, los artículos publicados en revistas indexadas en las bases de datos: *WOS*, *Scopus*, *Pubmed*, *Scielo* y *Cochrane*, los artículos publicados en los idiomas: inglés, portugués y español con las palabras claves como cierre de estoma, reversión de estoma, ileostomía, colostomía, Enfermería. En cuanto a los criterios de exclusión se tomaron en cuenta: los artículos que no fueran publicados en revistas indexadas en las bases de datos seleccionadas para la revisión integrativa, los artículos que no relacionaran en sus resultados o palabras claves la reversión de estoma y fueran de la fecha establecida.

La ecuación de búsqueda incluyó los términos según el DeCS con la siguiente estructura:

*“stoma closure” OR “stoma reversal” OR “stoma takedown” OR “ostomy closure” OR “colostomy closure” OR “colostomy reversal” OR “colostomy takedown and nursing”*

Durante la revisión y el rastreo, se evidenció que los estudios primarios correspondían a metaanálisis, ensayos clínicos aleatorizados y estudios multicéntricos en los cuales se incluían registros de estudios observacionales. Posterior al rastreo inicial, se crea un registro de datos y se relacionan los artículos encontrados en cada uno de los buscadores y los filtros utilizados para cada base de datos consultada.

Etapa N.º 3: Evaluación de datos

Para realizar una interpretación exhaustiva e imparcial de los datos de fuentes primarias las publicaciones incluidas fueron ordenadas, codificadas y clasificadas inicialmente por la base de datos y la matriz de análisis, con previa revisión por parte de pares externos. Se tuvieron en cuenta la base de datos bibliográfica, autor principal, año, elementos metodológicos generales y la extracción de la información que le proporcionaba a las categorías.

Etapa N.º 4: Análisis de datos

Específicamente para este caso, la reducción de datos implicó determinar un sistema de clasificación el cual se realizó mediante la lectura detallada de las publicaciones y la clasificación por subgrupos de acuerdo con la temática abordada. Se garantizó que no se repitieran las publicaciones incluidas y se sometieron a la herramienta de análisis, mediante el instrumento de lectura crítica de Critical Appraisal Skills Programme (CASpe) para revisión sistemática, estudios cualitativos, cuantitativos y mixtos.

Para los sesgos de selección se tuvo en cuenta la elaboración del PRISMA y se integraron los estudios que respondían al fenómeno, similar a un sesgo de clasificación de información. Para poder mitigar este riesgo, se desarrollaron matrices en Excel para organizar la información.

**Gráfico 1:**
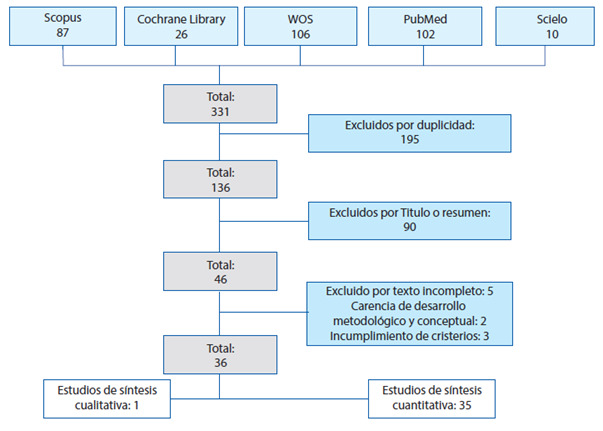
Declaración PRISMA

En la primera fase del rastreo, el número de publicaciones científicas indexadas en bases de datos correspondió a 331 artículos, de los cuales 195 fueron excluidos por duplicidad; de tal forma que se eligieron 136 publicaciones y, posteriormente, se depuraron 90 artículos que no mostraban relación con la temática en el título o resumen encontrando, para un total de 46 publicaciones. En seguida, se realizó el refinamiento de la búsqueda, encontrando 10 artículos que no evidenciaron en sus resultados intervenciones relacionadas con la reversión del estoma, lo que incumplía con el criterio de inclusión o de carencia en el desarrollo metodológico; por tanto, estos fueron excluidos. Así, se incluyeron 36 artículos en el análisis, distribuidos de la siguiente manera en las bases de datos seleccionadas: *WOS*: 15, *Pubmed*: 9, *Scopus*: 7, *Scielo*: 3 y *Cochrane:* 2.

La codificación de los artículos correspondió a un código asignado a cada artículo compuesto por una letra y un número, para expresar la base de datos a la que pertenecía y el número de publicación. Los códigos fueron asignados así: *WOS*: A, *Pubmed*: B, *Scopus*: C, *Scielo*: D y *Cochrane*: E.

## Resultados

**Tabla 1:** Publicaciones incluidas

Código / Titulo / Referencia	Año	Resultad o / Intervención
*A1 Stoma-site hernia after stoma reversal following rectal cancer resection*^*11*^	2020 Dinamarca	Factores de riesgo asociados a hernia incisional: obesidad, tabaquismo, edad, hipertensión arterial, diabetes e IMC > 30; No hay relación con el tipo de sutura usada para el cierre de la piel.
*A2 Early Versus Routine Stoma Closure in Patients With Colorectal Resection: A Meta-Analysis of 7 Randomized Controlled Trials*^*12*^	2020 China	El cierre temprano del estoma (4 semanas) es seguro y efectivo, con mayor riesgo de complicaciones en la herida.
*A3 Recurrence of rectal anastomotic leakage following stoma closure: assessment of risk factors*^*13*^	2019 Japón	La incidencia de fuga anastomótica es del 13%. Los factores predisponentes son: la anastomosis con sutura manual, la presencia de isquemia en el sitio de anastomosis, y cierre tardío.
*A4 Prophylactic mesh placement to avoid incisional hernias after stoma reversal: a systematic review and meta-analysis*^*14*^	2019 Holanda	El uso de una malla profiláctica reduce aparentemente el riesgo de hernia incisional después de la reversión del estoma, sin diferencia significativa en el desarrollo de ISO.
*A5 Factors affecting the morbidity and mortality of diverting stoma closure: retrospective cohort analysis of twelve-year period*^*15*^	2019 Eslovenia	La reversión del estoma menor a 8 meses se asocia a una tasa más baja de complicaciones y es el único factor predictivo.
*A6 A protocol for skin closure after stoma reversal*^*16*^	2019 EEUU	Se propone la técnica de cierre lineal con reversión de bordes y reducción del espacio muerto en pacientes cuya medición del grosor del tejido celular subcutáneo sea < 4 cm; con mejor resultado cosmético y disminución en la tasa de ISO.
*A7 Quality of life following ostomy reversal with purse-string vs linear skin closure: a systematic review*^*17*^	2018 Italia	La calidad de vida y satisfacción del paciente no tiene diferencias significativas después de la primera semana postoperatoria, según el tipo de cierre de piel; aunque inicialmente es menor en el cierre subcuticular.
*A8 Purse-string skin closure versus linear skin closure techniques in stoma closure: a meta-analysis with trial sequential analysis of randomized trials*^*18*^	2018 Reino Unido	El cierre de piel circunferencial o subcuticular está asociado con menor riesgo de ISO y mayor satisfacción del paciente comparado con el cierre de piel lineal.
*A9 Purse-string closure versus conventional primary closure of wound following stoma reversal: Meta-analysis of randomized controlled trials*^*19*^	2018 Italia	La técnica de cierre de piel circunferencial en la reversión del estoma es un procedimiento que disminuye significativamente el riesgo de ISO en comparación con la técnica de cierre lineal.
*A10* ^*)*^ *Purse-string approximation vs. primary closure with a drain for stoma reversal surgery: results of a randomized clinical trial*^*20*^	2018 Japón	En el cierre de piel circunferencial el tiempo de cicatrización de la herida es mayor a 30 días, comprometiendo la satisfacción del paciente, aunque muestra una menor incidencia de ISO. Así mismo, demanda mayor tiempo de cuidado y puede generar aumento en el costo de insumos hospitalarios.
*A11 Considerations in Stoma Reversal*^*21*^	2017 EEUU	Consideraciones para la reversión del estoma: valoración digital del esfínter anal. No se recomienda el uso del enema de contraste, evaluar el cierre de la fuga anastomótica previo a la cirugía; sutura de anastomosis con grapas. Cierre de piel con técnica subcuticular. Se sugiere reversión de Hartmann por vía laparoscópica.
*A12 A Descriptive, Qualitative Study to Assess Patient Experiences Following Stoma Reversal After Rectal Cancer Surgery*^*22*^	2017 Suecia	Posterior a la cirugía de reversión, los pacientes requieren seguimiento regular por los profesionales de salud para evaluar y abordar los resultados funcionales e informar sobre tratamientos de complicaciones tardías, principalmente la incontinencia fecal.
*A13 Quality of Life and Timing of Stoma Closure in Patients With Rectal Cancer Undergoing Low Anterior Resection With Diverting Stoma: A Multicenter Longitudinal Observational Study*^*23*^	2016 Alemania	La presencia de un estoma genera un impacto negativo; afectando la calidad de vida de la persona, principalmente a nivel de relaciones interpersonales, el deseo sexual e imagen corporal.
*A14 Determinants of stoma reversal in rectal cancer patients who had an anterior resection between 2009 and 2012 in the English National Health Service*^*24*^	2016 Reino Unido	Las tasas de reversión del estoma depende de características propias del paciente como: edad, sexo, estado físico, comorbilidades, tipo de cirugía, estadio de la enfermedad y nivel socioeconómico.
*A15 When is the best time for temporary stoma closure in laparoscopic sphincter-saving surgery for rectal cancer? A study of 259 consecutive patients*^*25*^	2015 Francia	No hay beneficio en retrasar el cierre de estoma después de completar la quimioterapia adyuvante. Se recomienda antes de 90 días.
*B1 Timing of colostomy reversal following Hartmann's procedure for perforated diverticulitis*^*26*^	2020 Israel	Previo a la reversión del estoma deben evaluarse factores de riesgo como: edad, sexo y comorbilidades. En procedimientos de Hartmann, el cierre tardío se asocia con la contracción del muñón rectal, generando una anastomosis más complicada
*B2 Meta-analysis and single-center experience on the protective effect of negative suction drains on wound healing after stoma reversal*^*27*^	2019 Alemania	El uso de terapia de presión negativa es beneficioso en la prevención de complicaciones (seroma, hematoma, infección) en heridas secundarias a la reversión de ileostomía con cierre primario; principalmente en pacientes obesos.
*B3 The Safety of Outpatient Stoma Closure: on the Verge of a Paradigm Shift?*^*28*^	2018 EEUU	La reversión de la ostomía puede realizarse de manera ambulatoria en aquellos pacientes con menor riesgo de complicaciones, de acuerdo con la evaluación preoperatoria y control de comorbilidades.
*B4 Evaluation of risk factors for complications after colostomy closure*^*29*^	2018 Turquía	Entre los factores de riesgo asociados a complicaciones, se identificó que la diabetes se relaciona con aumento en la incidencia ISO y evisceración.
*B5 Comparing Surgical Site Infection and Scar Cosmesis Between Conventional Linear Skin Closure Versus Purse-string Skin Closure in Stoma Reversal A Randomized Controlled Tria*^*30*^	2018 India	El cierre de piel subcuticular presenta menor incidencia de ISO; así mismo, se asocia a menor tiempo de terapia antibiótica y mejora significativamente el resultado estético de la cicatriz y la satisfacción del paciente, con respecto al cierre lineal.
*B6 Study Protocol Evaluating the Use of Bowel Stimulation Before Loop Ileostomy Closure to Reduce Postoperative Ileus: A Multicenter Randomized Controlled Trial*^*31*^	2017 Canadá	Evalúa el efecto de la estimulación intestinal preoperatoria antes de la reversión de ileostomía para disminuir el riesgo de íleo posoperatorio.
*B7 Early closure of defunctioning stoma increases complications related to stoma closure after concurrent chemoradiotherapy and low anterior resection in patients with rectal cancer*^*32*^	2017 Taiwán	El tiempo óptimo de reversión del estoma es aproximadamente de 109 días posterior a la construcción de este y se identifica como un factor de riesgo independiente para el desarrollo de complicaciones.
*B8 Colostomy closure: risk factors for complications*^*33*^	2017 Brasil	El tiempo quirúrgico, transfusión sanguínea y el traslado a UCI son factores de riesgo asociados con complicaciones, con una tasa de morbilidad del 20%.
*B9 Morbidity of colostomy reversal*^*34*^	2016 Pakistán	La reversión de colostomía se asocia con una morbilidad del 41,6%; la complicación más común es la infección de la herida y hernia incisional; estas se relacionaron directamente con una estancia hospitalaria prolongada.
*C1 The Watford low anterior resection syndrome pathway for preAnd post-stoma reversal patients*^*35*^	2019 Reino Unido	Se describe un protocolo de preparación intestinal y se evalúa el riesgo de incontinencia fecal mediante un dispositivo de irrigación transanal en pacientes programados para reversión del estoma.
*C2 Reconstrucción de la continuidad digestiva tras cirugía de la diverticulitis aguda complicada. Estudio retrospectivo multicéntrico*^*36*^	2018 España	Las fallas en la reconstrucción de la continuidad digestiva en diverticulitis aguda se asocian con diversas variables como: el tipo de cirugía, estado inmunosupresión, puntaje ASA, inestabilidad hemodinámica en la primera cirugía, el sexo femenino, y la edad.
*C3 The vulkan technique: a novel ostomy-closure technique that reduces complications and operative times*^*37*^	2017 Alemania	Técnica semejante al cierre de piel subcuticular, caracterizada por la colocación de suturas consecutivas en 3 capas. En la primera, se incluye la aponeurosis del músculo recto abdominal para reducir el espacio potencial para la acumulación de líquido y disminución de la incidencia de ISO.
*C4 Identifying Patients Eligible for a Short Hospital Stay After Stoma Closure*^*38*^	2017 Francia	La reversión de ostomía puede considerarse como un procedimiento ambulatorio (estancia <5 días) a partir de criterios de elegibilidad de pacientes, según la evaluación preoperatoria.
*C5 Enhanced recovery after surgery (ERAS) protocol in stoma reversals*^*39*^	2017 Pakistán	La aplicación del protocolo ERAS disminuye las complicaciones perioperatorias en términos de estancia hospitalaria, resolución de íleo, infección del sitio operatorio y disminución de costos hospitalarios.
*C6 The impact of surgeon volume on colostomy reversal outcomes after Hartmann's procedure for diverticulitis*^*40*^	2016 EEUU	La experticia del cirujano se asocia a disminución en la mortalidad postoperatoria y reducción en los costos de atención médica.
*C7 Fast-Tracking Colostomy Closures*^*41*^	2015 India	La aplicación del protocolo ERAS en la reversión del estoma se asocia con la reducción de la estancia hospitalaria.
*D1 Italian guidelines for the surgical management of enteral stomas in adults*^*42*^	2019 Italia	Recomendaciones basadas en evidencia científica: cierre temprano (posterior 2 semanas) en pacientes sin complicaciones intraoperatorias (IIa); anastomosis intestinal con grapas (Ia); técnica de cierre de piel subcuticular (Ia), profilaxis antibiótica (Ic).
*D2 El estudio radiológico con contraste antes del cierre del estoma derivativo en el cáncer de recto no es necesario de forma rutinaria*^*43*^	2018 España	El estudio radiológico de la anastomosis colorrectal antes de la reconstrucción del tránsito tiene sensibilidad del 38,9% y especificidad del 95,9% y puede omitirse con seguridad en los pacientes sin sepsis pélvica ni íleo paralítico, tras la resección anterior de recto.
*D3 Effectiveness Between Early and Late Closure of Rectal Cancer Patients with Temporary Ileostomy: A Prospective Study Timing of closure of temporary ileostomy*^*44*^	2017 China	En pacientes con quimioterapia neoadyuvante, se recomienda un cierre tardío (posterior a 6 meses) hasta completar el tratamiento.
*E1 The effectiveness of negative-pressure wound therapy for wound healing after stoma reversal: a randomised control study (SR-PICO study)*^*45*^	2015 Filipinas	La aplicación de terapia de presión negativa en la herida abierta secundaria a la reversión de ostomía con técnica subcuticular reduce el tiempo de cicatrización.
*E2 A randomized controlled clinical trial comparing the outcomes of circunferential subcuticular wound approximation (CSWA) with conventional wound closure after stoma reversal*^*46*^	2015 Filipinas	El cierre subcuticular de piel se asocia a una menor tasa de ISO y mayor satisfacción del paciente con respecto al resultado estético.

Fuente: Elaboración propia (2020)

En la tabla anterior, se especifican los artículos seleccionados con su respectivo resultado, año y país. Con respecto al idioma, se evidenciaron 33 en idioma inglés, 2 español y 1 portugués. De acuerdo con los hallazgos encontrados en cada una de las piezas investigativas y el análisis de los artículos para extraer las intervenciones de Enfermería, los artículos se lograron clasificar por el nivel de evidencia.

**Tabla 2:** Nivel de evidencia

Nivel de evidencia		Frecuencia	Porcentaje
I	Revisión sistemática y metaanálisis de ensayos controlados aleatorios; guías clínicas basadas en revisiones sistemáticas o metaanálisis	7	19%
II	Uno o más ensayos controlados aleatorios	5	14%
III	Ensayo controlado (sin aleatorización)	1	3%
IV	Estudio de casos y controles o estudio de cohorte	6	17%
V	Revisión sistemática de estudios descriptivos y cualitativos	0	0%
VI	Estudio descriptivo o cualitativo único	17	47%
	Total	36	100%

Fuente: Adaptación de Melnyk, BM y Fineout-Overholt, E. (2015)

Específicamente, en los hallazgos expuestos, se evidenció la concentración de estudios con nivel VI, lo cual traduce que las publicaciones no tuvieron fuerte evidencia científica; sin embargo, es de resaltar que su alcance y metodologías empleadas también son necesarias en la práctica, puesto que estos estudios son de gran aporte en cuanto a las intervenciones para la reversión de la estoma. Es importante mencionar que no todas las preguntas de investigación pueden ser resueltas de la misma manera, teniendo en cuenta que cada uno de los niveles de evidencia dan respuesta a diferentes cuestiones.

### Categorías de reversión del estoma intestinal y definición de intervenciones de enfermería 

Para el análisis de los resultados, se tuvo en cuenta una serie de categorías generadas a partir de la lectura de los artículos que han sido seleccionados para esta revisión.

**Tabla 3:** Categorías de reversión del estoma intestinal

Periodo	Definición	Categoría	Artículo
Preoperatorio	Elementos que se deben considerar y evaluar previo al procedimiento quirúrgico, garantizando la preparación óptima del paciente.	Tiempo de reversión	^26^^,^^12^^,^^13^^,^^15^^,^^23^^,^^25^^,^^32^^,^^31^^,^^36^^,^^43^^,^^46^
		Valoración preoperatoriaPreparación intestinal	^22^^,^^14^^,^^38^^,^^33^^,^^39^^,^^41^
		Seguimiento de comorbilidades	^11^^,^^15^^,^^24^^,^^26^^,^^28^^,^^29^^,^^33^, ^38^
		Estudios complementarios	^19^^,^^40^^,^^41^, ^43^
Intraoperatorio	Actividades durante el período intraoperatorio orientadas a disminuir el riesgo de complicaciones	Profilaxis antibiótica	^41^^,^^42^
		Preparación de la piel	^16^
		Técnica quirúrgica	^12^^,^^16^^,^^13^^,^^21^^,^^27^^,^^42^^,^^43^
		Cierre de la pared abdominal	^16^^,^^17^^,^^18^^,^^19^^,^^20^^,^^27^^,^^30^^,^^35^^,^^37^^,^^40^^,^^41^^,^^46^
Posoperatorio	Intervenciones orientadas al tratamiento de complicaciones tempranas y tardías, así como la educación y entrenamiento del paciente en la restauración del tránsito intestinal	Cuidado de la herida Complicaciones tardías	^27^, ^30^^,^^41^^,^^42^^,^^45^
		Calidad de vida y satisfacción del paciente	^21^^,^^34^^,^^35^^,^^40^
		Cuidado de la herida Complicaciones tardías	^12^^,^^20^^,^^23^, ^30^, ^37^^,^^44^^,^^46^
		Educación al paciente	^22^^,^^23^^,^^25^^,^^40^^,^^46^

Fuente: Elaboración propia-análisis de categorías. (2020)

### Intervenciones de Enfermería en el preoperatorio

#### Categoría 1: Tiempo de reversión

Existen diferencias de criterio con respecto al tiempo para realizar la reversión del estoma intestinal^12^^,^^13^^,^^15^. Se ha mencionado que se debe dar una espera de seis meses desde la cirugía inicial para realizar la reconexión y se sugiere que, durante este tiempo, el paciente puede recuperar peso, mejorar la nutrición y disminuir la presencia de adherencias intestinales^26^^,^^46^. Por el contrario, otros estudios mencionan que realizar el procedimiento entre los tres y seis meses posteriores a la primera cirugía favorece la reversión de un procedimiento de *Hartmann* hasta con un 80% de éxito; esto debido a que se previene la atrofia del segmento distal rectal^32^^,^^46^ (Nivel de evidencia I).

#### Categoría 2: Valoración preoperatoria-Preparación intestinal

En primer lugar, se debe brindar información amplia al paciente sobre del procedimiento a realizar, la recuperación esperada, las posibles complicaciones y el abordaje oportuno^22^^,^^14^^,^^23^^,^^29^ (Nivel de evidencia IV).

Mantener el periodo de ayuno de mínimo seis horas para sólidos y dos horas para líquidos, previo a una reversión del estoma^33^^,^^39^^,^^41^ (Nivel de evidencia I).

Es necesaria la administración por vía oral de una carga de solución isotónica de carbohidratos de 100g la noche previa a la cirugía, con una segunda carga de 50g de dos a tres horas antes de iniciar el procedimiento. No se considera adecuado dar dicha carga a pacientes diabéticos, con gastroparesia o vaciamiento gástrico lento^38^^,^^39^ (Nivel de evidencia IV).

No se recomienda la preparación intestinal mecánica rutinaria en procedimientos de restauración del tránsito colónico^38^ (Nivel de evidencia II).

#### Categoría 3: Seguimiento a comorbilidades

Se recomienda dejar de fumar de cuatro a seis semanas antes de la cirugía (valoración previa) y suspender el consumo de alcohol previo al procedimiento de restauración del tránsito intestinal^11^^,^^24^^,^^33^ (Nivel de evidencia II).

Se debe realizar el control metabólico previo a la cirugía con valores de glicemia menor a 180mg/dL y HbA1c menor a 7^24^^,^^29^; dado que los estados de hiperglicemia se relacionan directamente con un aumento en el riesgo de complicaciones como la fuga anastomótica (Nivel de evidencia IV).

No hay que suspender el antihipertensivo previo al procedimiento y debe reiniciarse con la mayor brevedad posible posterior a la cirugía^26^^,^^33^ (Nivel de evidencia IV).

En pacientes con desnutrición proteico-calórica, pérdida de peso de más del 10% o albúmina sérica menor a 3,0g/dL, se debe optimizar el estado nutricional previo al procedimiento y se sugiere realizar una valoración por los servicios de Nutrición y Soporte Nutricional^15^^,^^43^ (Nivel de evidencia VI).

#### Categoría 4: Estudios complementarios

Verificar los exámenes preoperatorios, el seguimiento al hemograma, la función renal, las pruebas de coagulación, de electrolitos y de albúmina^39^^,^^40^^,^^43^ (Nivel de evidencia IV).

Se debe realizar el electrocardiograma, la radiografía de tórax y la valoración por cardiología en pacientes mayores de 50 años con antecedentes de: hipertensión arterial, diabetes o enfermedad coronaria, enfermedad cerebrovascular, insuficiencia cardiaca congestiva y, en caso de riesgo elevado, solicitar recomendaciones preoperatorias del especialista^39^^,^^43^ (Nivel de evidencia IV).

Es importante la realización de la colonoscopía a través de colostomía para evaluar la funcionalidad intestinal y descartar el riesgo de fuga anastomótica^33^ (Nivel de evidencia IV).

En caso de antecedente de ostomías por neoplasia colorrectal, se recomienda realizar TAC de abdomen y de pelvis con medio de contraste como parte del seguimiento y descartar la enfermedad neoplásica activa^19^^,^^41^ (Nivel de evidencia IV).

Se debe realizar el estudio radiológico de la anastomosis colorrectal antes de la reversión del estoma^43^ (Nivel de evidencia IV).

### Intervenciones de Enfermería en el intraoperatorio

#### Categoría 5: Profilaxis antibiótica

La profilaxis antibiótica con Cefalotina 1g IV + Metronidazol 500mg IV se debe administrar una hora antes de la incisión, con ajuste de la dosis en función del peso^41^^-^^42^ (Nivel de evidencia I).

No se recomienda la administración profiláctica de antibióticos después del cierre de la incisión y no existe beneficio de prolongar las dosis por más de 24 horas^42^ (Nivel de evidencia I).

#### Categoría 6: Preparación de la piel

1.Se deben utilizar soluciones con alcohol como primera línea (clorhexidina con alcohol), para el lavado del sitio quirúrgico y preparación de la piel previo a la incisión, así como evitar la depilación del vello^16^ (Nivel de evidencia II).

#### Categoría 7: Técnica quirúrgica

Es beneficioso realizar el procedimiento quirúrgico de reversión del estoma mediante técnica laparoscópica, condicionado a la experticia del cirujano, dado que se relaciona con mejores resultados, menor índice de complicaciones y del tiempo de estancia hospitalaria^13^^,^^21^^,^^27^^,^^42^ (Nivel de evidencia IV).

En la anastomosis intestinal, se recomienda el uso de grapas dado que se relaciona con menor tiempo quirúrgico comparado con la anastomosis con sutura manual^42^ (Nivel de evidencia I).

Se recomienda mantener la normotermia intraoperatoria y evitar la hipotermia, pues puede provocar vasoconstricción periférica relacionada con el aumento en la tasa de infección del sitio operatorio y fuga anastomótica^21^ (Nivel de evidencia II).

El uso de una malla profiláctica reduce aparentemente el riesgo de hernias incisionales después de la reversión del estoma, sin diferencia significativa en el desarrollo de ISO^12^^,^^16^ (Nivel de evidencia I).

#### Categoría 8: Cierre de la pared abdominal

Es importante realizar el cambio de guantes antes del cierre de la piel para prevenir infecciones del sitio quirúrgico^40^^,^^41^ (Niel de evidencia I).

En el cierre de piel de la herida secundaria a la ostomía, se debe hacer uso de la técnica subcuticular circunferencial, dado que presenta menor tasa de infección del sitio operatorio con respecto a otras técnicas quirúrgicas^17^^,^^18^^,^^19^^,^^27^^,^^30^ (Nivel de evidencia I).

Debe realizarse la prueba neumática de una anastomosis colorrectal, la cual permite identificar fugas de manera inmediata con la posibilidad de reparar el defecto o hacer una o una ostomía derivativa en el caso de una prueba positiva^31^ (Nivel de evidencia I).

### Intervenciones en el posoperatorio

#### Categoría 9: Cuidado de la herida

La aplicación de la terapia de presión negativa en la herida abierta secundaria a la reversión del estoma con técnica subcuticular, reduce el tiempo de cicatrización^41^^,^^42^^,^^45^ (Nivel de evidencia II).

#### Categoría 10: Complicaciones tardías

Posterior a la cirugía de reversión, los pacientes requieren seguimiento regular por los profesionales de salud para evaluar y abordar los resultados funcionales e informar sobre los tratamientos de complicaciones tardías, principalmente de la incontinencia fecal^21^^,^^34^^,^^40^ (Nivel de evidencia VI).

La experticia del cirujano se asocia a la disminución en la mortalidad postoperatoria y la reducción en los costos de atención médica^40^ (Nivel de evidencia IV).

#### Categoría 11: Calidad de vida y satisfacción del paciente

En el cierre de piel circunferencial, el tiempo de cicatrización de la herida es mayor, lo que compromete la satisfacción del paciente; asimismo, demanda mayor tiempo de cuidado y puede generar el aumento en el costo de insumos hospitalarios^12^^,^^20^^,^^30^^,^^37^ (Nivel de evidencia II).

La calidad de vida y la satisfacción del paciente no tienen diferencias significativas después de la primera semana postoperatoria con respecto al tipo de cierre de piel usado; sin embargo, el índice de satisfacción es menor con el cierre subcuticular inicialmente relacionado con la herida abierta, secundario a las implicaciones del tratamiento^17^^,^^44^^,^^46^ (Nivel de evidencia I).

#### Categoría 12: Educación al paciente

Es recomendable dar seguimiento al paciente intervenido por reversión del estoma a los 30 días de su cirugía^22^^,^^23^ (Nivel de evidencia IV).

Debe evaluarse el riesgo de incontinencia fecal y brindarse información clara sobre los síntomas y tratamientos relacionados, dado que es la complicación más común en este tipo de procedimientos^22^^,^^45^^,^^46^ (Nivel de evidencia V).

## Discusión

De acuerdo con las intervenciones encontradas, es importante resaltar el tiempo de reversión del estoma, en compararon con el tiempo convencional (de ocho a doce semanas desde la cirugía inicial) con el cierre temprano (dentro de las cuatro semanas desde la cirugía inicial). La mayoría de los datos provienen de pacientes con ileostomía en asa sometidos a cirugía rectal por cáncer. Los estudios coinciden en que no existen diferencias significativas en cuanto a fugas anastomóticas con relación al momento de reversión del estoma.

En un ensayo clínico aleatorizado, el cierre temprano de la ileostomía (en el octavo día posoperatorio) dio mejores resultados con menos obstrucciones del intestino delgado, así como tasas más bajas de complicaciones médicas y una estancia hospitalaria más corta; mientras que hubo tasas más bajas de complicaciones de la herida en el grupo de cierre tardío (más de doce semanas desde la cirugía índice)^46^. Así mismo, los resultaros son similares con el ensayo EASY que encontró una tasa más baja de complicaciones en ileostomías y colostomías después de la cirugía con un seguimiento de doce meses en el grupo temprano realizado entre ocho y trece días desde la cirugía inicial^47^.

Se encontraron resultados similares en cuatro ensayo clínico aleatorizado, sin encontrar diferencias en términos de fuga o estenosis anastomótica, complicaciones postoperatorias, duración de la estancia hospitalaria y duración de la operación^48^. De igual forma, en un metaanálisis del año 2020 se compara la seguridad y viabilidad del cierre temprano del estoma (cuatro semanas) con respecto al tiempo de cierre rutinario (ocho semanas). Dentro de los resultados, se encuentra que no hay diferencias significativas con respecto a complicaciones; sin embargo, el cierre temprano se asocia con una menor tasa de obstrucción intestinal y mayor riesgo de complicaciones en la herida quirúrgica como infección del sitio operatorio^49^.

Paralelamente, en cuanto a la técnica quirúrgica, estudios recomiendan que se realice vía laparoscópica, dado que muestra beneficios con respecto a la técnica abierta^46^. Esta vía es útil según la selección de pacientes con una baja morbilidad (16,6%) y mortalidad (0,7%), y una tasa de conversión exitosa del 11,7%, así como para una menor tasa de infecciones de sitio operatorio y menor necesidad de reintervención, además de la disminución en días de estancia hospitalaria (cinco versus seis días), lo que evidencia una significancia estadística en el procedimiento mínimamente invasivo al tener una mortalidad y tiempo quirúrgico similares^49^.

La experiencia del cirujano es también un factor determinante para la prevención de complicaciones mayores en una reversión de ostomía, pues esta es un factor de riesgo independiente de la fuga anastomótica^46^. Evidencia de esto es la realización de un estudio con 10.487 pacientes sometidos a reversión de un procedimiento de *Hartmann* para determinar la relación entre el cirujano y los resultados. Se identificó que aquellos cirujanos que tienen un alto volumen de pacientes obtuvieron mejores resultados postquirúrgicos^50^. De igual forma, los protocolos de ERAS (*Enhanced Recovery After Surgery*) tienen considerables ventajas con resultados mejores en comparación con el sistema convencional^41^, así como el *fast-track* se ha asociado con la disminución en la estancia hospitalaria sin aumento en la readmisión, un menor porcentaje de íleo postoperatorio y la disminución en las complicaciones postoperatorias, con resultados equiparables y mejores en comparación con los protocolos convencionales^44^^,^^50^.

Con respecto a la preparación intestinal, un metaanálisis de *Cochrane*, con 18 estudios aleatorizados controlados en 5.805 pacientes, comparó la preparación mecánica intestinal con la no preparación, y la preparación mecánica intestinal con enemas rectales^41^^,^^51^. Se determinó que no existe evidencia estadísticamente significativa de que los pacientes se beneficien de una preparación mecánica intestinal ni de enemas rectales en términos de resultados, mortalidad, incidencia de fugas anastomóticas, necesidad de reintervención e infecciones del sitio quirúrgico. Dicho estudio concluyó que en la cirugía colónica, es seguro omitir la preparación mecánica sin afectar la tasa de complicaciones. A nivel de cirugía rectal, los estudios sugieren que la preparación mecánica sea utilizada de forma selectiva pese a que no se determinó efecto significativo con dicha medida^52^.

## Conclusiones

Las intervenciones de enfermería en la reversión del estoma intestinal son de gran relevancia para el profesional de enfermería y los estomaterapeutas, en el preoperatorio; valoración de enfermería, verificación de exámenes complementarios, información a la persona por parte del equipo quirúrgico y la preparación intestinal. Con respecto, al intraoperatorio; profilaxis antibiótica, preparación de la piel, verificación de la técnica quirúrgica y en el posoperatorio; educación al paciente, afrontamiento a los cambios intestinales y emocionales, el cuidado de la herida, valoración de complicaciones tempranas y tardías. Lo anterior dado que pueden presentar problemas severos de la función intestinal, incluido el aumento de la frecuencia de las deposiciones y la incapacidad para anticipar o confiar en la función intestinal; sin embargo, se subestima la educación posterior al cierre del estoma.

Es prioritario para el paciente estar informado y seguro de que sus síntomas intestinales son normales para favorecer el afrontamiento de estos; del mismo modo, es imperativo el apoyo de sus familiares y amigos durante este proceso de adaptación a su nueva imagen y función intestinal, fase en la cual la mayoría realiza un abordaje satisfactorio al usar estrategias que aprendieron sobre la dieta y la medicación antes de la reversión del estoma para tratar de desafiar las restricciones de su nueva normalidad .

Enfermería cumple un papel importante en el procedimiento de la reversión del estoma, no solo por los cuidados físicos y la educación que brinda al paciente, sino también en las intervenciones aplicables al contexto social, mental, sexual y emocional que deben sercontemplados por el impacto que puedan tener en el individuo. No solo se debe tener en cuenta un enfoque fisiológico y funcional del paciente, también es importante considerar su contexto psicoemocional, el cual debe ser analizado por el personal de salud, dado que el paciente con cierre ostomal experimenta cambios en su estructura mental que lo obligan a sufrir una desadaptación al estilo que vida que había formado como parte de su cotidianidad.

Se recomienda realizar estudios relacionados con el rol de Enfermería en la reversión del estoma intestinal. Como se ha demostrado en esta revisión, desde el área disciplinar se desarrollan intervenciones específicas en cada una de las categorías descritas y se hace necesario mediante evidencia científica cualificar y medir el impacto de estas actividades en la recuperación óptima de la persona y la disminución de complicaciones relacionadas con el procedimiento.

## References

[1] Hendren S, Hammond K, Glasgow SC, Perry WB, Buie WD, Steele SR (2015). Clinical practice guidelines for ostomy surgery. Dis Colon Rectum..

[2] Pine J, Stevenson L, On J (2020). Intestinal stomas. Surg - Oxford Int.

[3] Iqbal F, Kujan O, Bowley DM, Keighley MRB, Vaizey CJ (2016). Quality of Life After Ostomy Surgery in Muslim Patients: A Systematic Review of the Literature and Suggestions for Clinical Practice. J Wound Ostomy Cont Nurs..

[4] Fazekas B, Fazekas B, Hendricks J, Smart N, Arulampalam T (2017). The incidence of incisional hernias following ileostomy reversal in colorectal cancer patients treated with anterior resection. Ann R Coll Surg Engl..

[5] Yin T-C, Tsai H-L, Yang P-F, Su W-C, Ma C-J, Huang C-W (2017). Early closure of defunctioning stoma increases complications related to stoma closure after concurrent chemoradiotherapy and low anterior resection in patients with rectal cancer. World J Surg Oncol.

[6] Waterland P, Goonetilleke K, Naumann DN, Sutcliff M, Soliman F (2015). Defunctioning Ileostomy Reversal Rates and Reasons for Delayed Reversal: Does Delay Impact on Complications of Ileostomy Reversal? A Study of 170 Defunctioning Ileostomies. J Clin Med Res..

[7] Sparreboom CL, Wu Z-Q, Ji J-F, Lange JF (2016). Integrated approach to colorectal anastomotic leakage: Communication, infection and healing disturbances. World J Gastroenterol..

[8] Sousa MJ de, Andrade SS da C, Brito KKG de, Matos SD de O, Coêlho HFC, Oliveira SH dos S (2016). Sociodemographic and clinical features and quality of life in stomized patients. Journal of Coloproctology.

[9] Ye X., He D., Zhao J., Lei Y., Yao Q. (2019). Wang H. Application value of nursing intervention combined with early nutritional support in preventive stoma reversion of low rectal cancer. Oncol Lett..

[10] Whittemore R (2005). Combining evidence in nursing research: methods and implications. Nurs Res..

[11] Mongelard K, Mynster T, Jensen KK (2020). Stoma-site hernia after stoma reversal following rectal cancer resection. Dan Med J..

[12] Guo Y, Luo Y, Zhao H, Bai L, Li J, Li L (2020). Early Versus Routine Stoma Closure in Patients With Colorectal Resection: A Meta-Analysis of 7 Randomized Controlled Trials. Surg Innov..

[13] Kitaguchi D, Nishizawa Y, Sasaki T, Tsukada Y, Ikeda K, Ito M (2019). Recurrence of rectal anastomotic leakage following stoma closure: assessment of risk factors. Colorectal Disease..

[14] Van den Hil LCL, Van Steensel S, Schreinemacher MHF, Bouvy ND (2019). Prophylactic mesh placement to avoid incisional hernias after stoma reversal: a systematic review and meta-analysis. Hernia.

[15] Krebs B, Ivanecz A, Potrc S, Horvat M (2019). Factors affecting the morbidity and mortality of diverting stoma closure: retrospective cohort analysis of twelve-year period. Radiol Oncol..

[16] Pemmaraju VT, Lansing SS, Husain S (2020). A protocol for skin closure after stoma reversal. Tech Coloproctol..

[17] Rausa E, Kelly ME, Sgroi G, Lazzari V, Aiolfi A, Cavalcoli F (2019). Quality of life following ostomy reversal with purse-string vs linear skin closure: a systematic review. Int J Colorectal Dis..

[18] Hajibandeh S, Hajibandeh S, Rehman S, Zadeh RA (2019). Purse-string skin closure versus linear skin closure techniques in stoma closure: a meta-analysis with trial sequential analysis of randomised trials. Br J Surg..

[19] Rondelli F, Franco L, Balzarotti RC, Ceccarelli G, Becattini C, Bugiantella W (2018). Purse-string closure versus conventional primary closure of wound following stoma reversal: Meta-analysis of randomized controlled trials. Int J Surg..

[20] Amano K, Ishida H, Kumamoto K, Okada N, Hatano S, Chika N (2019). Purse-string approximation vs. primary closure with a drain for stoma reversal surgery: results of a randomized clinical trial. Surg Today.

[21] Sherman KL, Wexner SD (2017). Considerations in Stoma Reversal. Clin Colon Rectal Surg..

[22] Reinwalds M, Blixter A, Carlsson E (2017). A Descriptive, Qualitative Study to Assess Patient Experiences Following Stoma Reversal After Rectal Cancer Surgery. Ostomy Wound Manage.

[23] Herrle F, Sandra-Petrescu F, Weiss C, Post S, Runkel N, Kienle P (2016). Quality of Life and Timing of Stoma Closure in Patients With Rectal Cancer Undergoing Low Anterior Resection With Diverting Stoma: A Multicenter Longitudinal Observational Study. Dis Colon Rectum..

[24] Kuryba AJ, Scott NA, Hill J, van der Meulen JH, Walker K (2016). Determinants of stoma reversal in rectal cancer patients who had an anterior resection between 2009 and 2012 in the English National Health Service. Colorectal Disease..

[25] Figueiredo MN, Mège D, Maggiori L, Ferron M, Panis Y (2015). When is the best time for temporary stoma closure in laparoscopic sphincter-saving surgery for rectal cancer? A study of 259 consecutive patients. Tech Coloproctol..

[26] Gröne J. (2019). Tiempo y técnica de reubicación de la ostomía, teniendo en cuenta las complicaciones de la ostomía anteriores y posteriores. Coloproctología.

[27] Neumann P-A, Reischl S, Berg F, Jäger C, Friess H, Reim D (2020). Meta-analysis and single-center experience on the protective effect of negative suction drains on wound healing after stoma reversal. Int J Colorectal Dis..

[28] Taylor JP, Stem M, Chen SY, Yu D, Fang SH, Gearhart SL (2019). The Safety of Outpatient Stoma Closure: ¿on the Verge of a Paradigm Shift?. J Gastrointest Surg..

[29] Goret NE, Goret CC, Cetin K, Agachan AF (2019). Evaluation of risk factors for complications after colostomy closure. Ann Ital Chir..

[30] Sureshkumar S, Jubel K, Ali MS, Vijayakumar C, Amaranathan A, Sundaramoorthy S (2018). Comparing Surgical Site Infection and Scar Cosmesis Between Conventional Linear Skin Closure Versus Purse-string Skin Closure in Stoma Reversal - A Randomized Controlled Trial. Cureus.

[31] Garfinkle R, Trabulsi N, Morin N, Phang T, Liberman S, Feldman L (2017). Study protocol evaluating the use of bowel stimulation before loop ileostomy closure to reduce postoperative ileus: a multicenter randomized controlled trial. Color Dis Off J Assoc Coloproctology Gt Britain Irel..

[32] Yin TC, Tsai HL, Yang PF, Su WC, Ma CJ, Huang CW (2017). El cierre temprano del estoma disfuncional aumenta las complicaciones relacionadas con el cierre del estoma después de la quimiorradioterapia concurrente y la resección anterior baja en pacientes con cáncer de recto. World J Surg Onc..

[33] Fonseca AZ, Uramoto E, Santos-Rosa OM, Santin S, Ribeiro MJ (2017). Colostomy closure: risk factors for complications. Arq Bras Cir Dig..

[34] Khan S, Alvi R, Awan Z, Haroon N (2016). Morbidity of colostomy reversal. J Pak Med Assoc..

[35] Sumner D, Collins B (2019). The Watford low anterior resection syndrome pathway for pre- And post-stoma reversal patients. Gastrointest Nurs.

[36] Roig J, Salvador A, Frasson M, García-Mayor L, Espinosa J, Roselló V (2018). Reconstrucción de la continuidad digestiva tras cirugía de la diverticulitis aguda complicada. Estudio retrospectivo multicéntrico. Cirugía Española.

[37] Krenzien F, Benzing C, Harders F, Junghans T, Rasim G, Bothe C (2017). The vulkan technique: A novel ostomy-closure technique that reduces complications and operative times. Arq Bras Cir Dig..

[38] Sabbagh C, Cosse C, Rebibo L, Hariz H, Dhahri A, Regimbeau JM (2018). Identifying Patients Eligible for a Short Hospital Stay After Stoma Closure. J Invest Surg..

[39] Pirzada MT, Naseer F, Haider R, Haider J, Ahmed MJ, Alam SN (2017). Enhanced recovery after surgery (ERAS) protocol in stoma reversals. J Pak Med Assoc..

[40] Aquina CT, Probst CP, Becerra AZ, Hensley BJ, Iannuzzi JC, Noyes K (2016). The impact of surgeon volume on colostomy reversal outcomes after Hartmann’s procedure for diverticulitis. Surgery.

[41] Nanavati AJ, Prabhakar S (2015). Fast-Tracking Colostomy Closures. Indian J Surg..

[42] Ferrara F, Parini D, Bondurri A, Veltri M, Barbierato M, Pata F (2019). Italian guidelines for the surgical management of enteral stomas in adults. Tech Coloproctol..

[43] Climent-Agustín M, Pascual M, Alonso S, Salvans S, Gil M., Grande L (2018). El estudio radiológico con contraste antes del cierre del estoma derivativo en el cáncer de recto no es necesario de forma rutinaria. Cirugía Española.

[44] Zhen L, Wang Y, Zhang Z, Wu T, Liu R, Li T (2017). Effectiveness between early and late temporary ileostomy closure in patients with rectal cancer: A prospective study. Curr Probl Cancer..

[45] Kim S, Kang SI (2020). The effectiveness of negative-pressure wound therapy for wound healing after stoma reversal: a randomised control study (SR-PICO study). Trials.

[46] Lopez MPJ, Melendres MFA, Maglangit SACA, Roxas MFT, Monroy HJ, Crisostomo AC (2015). Un ensayo clínico controlado aleatorio que compara los resultados de la aproximación circunferencial de la herida subcuticular (CSWA) con el cierre convencional de la herida después de la reversión del estoma. Tech Coloproctol..

[47] Park J, Danielsen AK, Angenete E, Bock D, Marinez AC, Haglind E (2018). Quality of life in a randomized trial of early closure of temporary ileostomy after rectal resection for cancer (EASY trial). Br J Surg..

[48] Farag S, Rehman S, Sains P, Baig MK, Sajid MS (2017). Early vs delayed closure of loop defunctioning ileostomy in patients undergoing distal colorectal resections: an integrated systematic review and meta-analysis of published randomized controlled trials. Colorectal Disease..

[49] Horesh N, Lessing Y, Rudnicki Y, Kent I, Kammar H, Ben-Yaacov A (2020). Timing of colostomy reversal following Hartmann’s procedure for perforated diverticulitis. J Visc Surg..

[50] Arkenbosch J, Miyagaki H, Kumara HMCS, Yan X, Cekic V, Whelan RL (2015). Efficacy of laparoscopic-assisted approach for reversal of Hartmann’s procedure: results from the American College of Surgeons National Surgical Quality Improvement Program (ACS-NSQIP) database. Surg Endosc..

[51] Van Rooijen SJ, Huisman D, Stuijvenberg M, Stens J, Roumen RMH, Daams F (2016). Intraoperative modifiable risk factors of colorectal anastomotic leakage: Why surgeons and anesthesiologists should act together. Int J Surg..

[52] Kiran RP, Murray ACA, Chiuzan C, Estrada D, Forde K. (2015). Combined preoperative mechanical bowel preparation with oral antibiotics significantly reduces surgical site infection, anastomotic leak, and ileus after colorectal surgery. Ann Surg..

